# Association between angiotensin-converting enzyme (ACE) gene I/D polymorphism with the risk of knee OA: A systematic review, meta-analysis, and meta-regression

**DOI:** 10.12688/f1000research.140233.1

**Published:** 2024-02-26

**Authors:** M. Nasser Mustari, Muh. Nasrum Massi, Muh. Andry Usman, Agussalim Bukhari, Irfan Idris, Alfian Zainuddin, Endy Adnan, Syakib Bakri, Mizwar Hatta, Haerani Rasyid, Achmad Fikry, Audrey Suryani Soetjipto

**Affiliations:** 1 Division of Orthopaedic and Traumatology, Department of Surgery, Faculty of Medicine, Hasanuddin University, Makassar, South Sulawesi, Indonesia; 2 Department of Clinical Microbiology, Faculty of Medicine, Hasanuddin University, Makassar, South Sulawesi, Indonesia; 3 Division of Orthopaedic and Traumatology, Department of Orthopaedic and Traumatology,, Hasanuddin University, Makassar, South Sulawesi, Indonesia; 4 Department of Clinical Nutrition, Faculty of Medicine, Hasanuddin University, Makassar, South Sulawesi, Indonesia; 5 Department of Physiology, Faculty of Medicine, Hasanuddin University, Makassar, South Sulawesi, Indonesia; 6 Department of Public Health, Faculty of Medicine, Hasanuddin University, Makassar, South Sulawesi, Indonesia; 7 Division of Rheumatology, Department of Internal Medicine, Hasanuddin University, Makassar, South Sulawesi, Indonesia; 8 Division of Kidney and Hypertension, Department of Internal Medicine, Hasanuddin University, Makassar, South Sulawesi, Indonesia; 9 Specialty & Research Laboratory, The Prodia Education and Research Institute, Jakarta City, Jakarta, Indonesia; 10 Department of Internal Medicine, Faculty of Medicine, Hasanuddin University, Makassar, South Sulawesi, Indonesia

**Keywords:** ACE, genetic models, polymorphism, osteoarthritis, knee

## Abstract

**Background:**

Previous studies have linked genetics to knee osteoarthritis. Angiotensin-converting enzyme (ACE) gene I/D polymorphism may cause OA. However, evidence remains inconsistent. This study examines knee OA risk and ACE gene I/D polymorphism.

**Methods:**

We explored Europe PMC, Medline, Scopus, and Cochrane Library using keywords. Three assessment bias factors were assessed using the Newcastle-Ottawa Scale (NOS). Criteria for inclusion: (1) Split the study population into knee OA patients and healthy controls; (2) Analysed the ACE gene I/D polymorphism; (3) Case-control or cross-sectional surveys. Studies with non-knee OA, incomplete data, and no full-text were excluded. The odds ratio (OR) and 95% confidence intervals (95% CI) were calculated using random-effect models.

**Results:**

A total of 6 case-control studies consist of 1,226 patients with knee OA and 1,145 healthy subjects as controls were included. Our pooled analysis revealed that a significant association between ACE gene I/D polymorphism and risk of knee OA was only seen in the dominant (DD + ID vs. II) [OR 1.69 (95% CI 1.14 – 2.50), p = 0.009, I2 = 72%], and ID vs. II [OR 1.37 (95% CI 1.01– 1.86), p = 0.04, I2 = 43%] genotype models. Other genotype models, including recessive (DD vs. ID + II), alleles (D vs. I), DD vs. ID, and DD vs. II models did not show a significant association with knee OA risk. Further regression analysis revealed that ethnicity and sex may influence those relationships in several genotype models.

**Conclusions:**

Dominant and ID vs. II ACE gene I/D polymorphism models increased knee OA risk significantly. More research with larger samples and different ethnic groups is needed to confirm our findings. After ethnicity subgroup analysis, some genetic models in our study showed significant heterogeneities, and most studies are from Asian countries with Asian populations, with little evidence on Arabs.

## Introduction

Osteoarthritis (OA) of the knee is the most common type of arthritis involving the knee joint (besides rheumatoid arthritis, post-traumatic arthritis, etc). ^1^ This disease is well-recognized as a major public health problem. ^1^ Epidemiologically, it is estimated that there were around 654.1 million people in the world who experience knee OA in 2020 with a global prevalence of 22.9% in individuals aged 40 years and over. ^2^ Knee OA is also one of the main causes of individual dysfunction/disability which may reduce the quality of life. ^2^ It is estimated that the burden of knee OA will continue to increase, along with increasing age and people with obesity. ^2^

In knee OA, there is a disintegration of the cartilage structure of the knee joint, which becomes softer and damaged, accompanied by imperfect growth of new cartilage and the formation of osteophytes around the joint. ^1^^,^^3^ Apart from increasing age and obesity, joint trauma and excessive workload on the joints are also risk factors for knee OA. ^2^^,^^3^ Researchers also found that genetic factors in the form of polymorphism in several genes also play an important role in increasing the risk of knee OA in certain individuals. ^2^^,^^3^ Previous research has shown that genetic factors contribute to 35-65% of a person’s overall risk of developing knee OA. ^4^

Angiotensin-converting enzyme (ACE) is a membrane-bound enzyme that catalyzes the conversion of angiotensin I to angiotensin II, a potent vasoconstrictor. ^5^ ACE also metabolizes bradykinin, a strong vasodilator, to its inactive form, namely bradykinin 1-5. ^5^ As an inflammatory vasodilator, bradykinin has an important role in the generation of pain, swelling, and inflammation through its receptors, mainly bradykinin receptor B1 (BKB1). ^6^^,^^7^ Bradykinin can promote cartilage degradation and inhibit the synthesis of cartilage proteoglycans and type II collagen which is the main components of the extra-cellular matrix (ECM) in articular cartilage. ^6^^,^^7^ Considering the important role of ACE in the renin-angiotensin system (RAS) and inactivation of bradykinin, some evidence suggests the possibility of ACE polymorphism in causing OA. ^8^ The ACE gene is located on chromosome 17 and contains a polymorphism based on the presence (insertion, I) or absence (deletion, D) of intron 16, of a 287 bp ALU repeat sequence; resulting in three genotypes: DD and II homozygotes and ID heterozygotes. ^9^^,^^10^ ACE levels in plasma were found to be highest in individuals with the DD genotype and lowest in individuals with the II genotype. ^9^^,^^10^

Unfortunately, previous studies still showed conflicting results on the relationship between ACE gene I/D polymorphism and the risk of knee OA. In their study of the Korean population, Hong SJ et al. ^11^ demonstrated that ACE gene I/D polymorphism, especially the I allele, is associated with early onset and radiographically severe knee OA. On the other hand, Shehab DK et al. ^12^ found no association between ACE gene I/D polymorphism genotype and primary knee OA in Kuwaiti patients. Therefore, this systematic review and meta-analysis aims to summarize evidence regarding the relationship between ACE gene I/D polymorphism and the risk of knee OA.

## Methods

### Eligibility criteria

This review was written based on the guidelines from the PRISMA statement. ^13^ We selected the literature for inclusion in this review if it met the following inclusion criteria: (1) The population of the study was divided into two groups, namely those with knee OA and healthy controls (without knee OA); (2) Analysed the ACE gene I/D polymorphism, which may be in the form of major (I) and minor (D) alleles as well as the distribution of genotype variations (DD, ID, and II) in both groups of patients; (3) Case-control studies or cross-sectional surveys. Meanwhile, literature in which one or more of the following exclusion criteria were fulfilled were removed from this review: (1) Studies involving OA other than knee; (2) Incomplete data; (3) Not available in the full-text form; (4) Studies beside case-control and cross-sectional.

### Literature search and study selection

A systematic literature search on 4 databases: Europe PMC, Medline, Scopus, and the Cochrane Library was conducted by two independent authors from the date of inception until January 12 ^th^, 2023. The search was limited to English-language literature only. We used the following keyword combinations to elicit relevant literature: “(knee osteoarthritis OR OA knee OR gonarthrosis OR osteoarthritis genu OR OA genu) AND (Angiotensin Converting Enzyme OR Angiotensin I-Converting Enzyme OR ACE OR Peptidyl-Dipeptidase A OR Dipeptidyl Peptidase A OR Kininase A OR Kininase II) AND (polymorphism OR insertion/deletion OR I/D)”. The process of identifying relevant literature through titles/abstract screening and duplicate removal was carried out by the same two authors. If the articles passed the screening process, they were evaluated in full-text format to match the eligibility criteria. All discrepancies in this review were resolved through discussion.

### Data extraction and quality assessment

The data extraction and tabulation into Microsoft Excel 2019 were carried out by two independent authors. The data extracted were as follows: author’s name, year of publication, country, number of samples, baseline characteristics of study participants, and distribution of ACE genotype variations or alleles in each patient group.

The same two authors also performed a risk of bias assessment of the included case-control studies using the appropriate tool. We used the Newcastle-Ottawa Scale (NOS) which covers three aspects of assessment: (1) selection of cases and appropriate controls; (2) comparability between two groups of participants; (3) measurement of the exposure. ^14^ The results of the evaluation using this tool were in the form of numbers from 0 to 9 where studies with a total score of ≥7 were categorized as “good” quality. ^14^

### Statistical analysis

Dichotomous variable outcomes were computed in the form of odds ratio (OR) along with 95% confidence intervals (95% CI) using the Mantel-Haenszel formula to compare the ACE gene I/D polymorphism between the two groups of patients. Random-effect models were chosen in this review because of the consideration that significant heterogeneity was expected due to differences in the population characteristics. Three common genetic models (dominant, recessive, and allele type) and three additional genotype models comparison (DD vs. DI, DD vs. II, and DI vs. II) were chosen to comprehensively assess the relationship between ACE gene I/D polymorphism and risk of knee OA. In this review, we used the I-squared (I ^2^) statistic to assess the heterogeneity between studies where I ^2^ values of ≤25%, 26 – 50%, and >50% were categorized as low, moderate, and high heterogeneity, respectively. ^15^ Meta-regression with a random-effects model was performed using a restricted-maximum likelihood for pre-specified variables including ethnicity, sample size, age, sex, BMI, Kellgren-Lawrence grade, and duration of OA to see the interaction effect between ACE gene I/D polymorphism, and these variables in influencing the risk of knee OA. A publication bias analysis was performed when there were more than 10 studies on each outcome of interest. All of these statistical analyses were carried out using an application from the Cochrane Collaboration, namely Review Manager 5.4 and Comprehensive Meta-Analysis version 3.

## Results

### Study selection and characteristics

**Figure 1.**
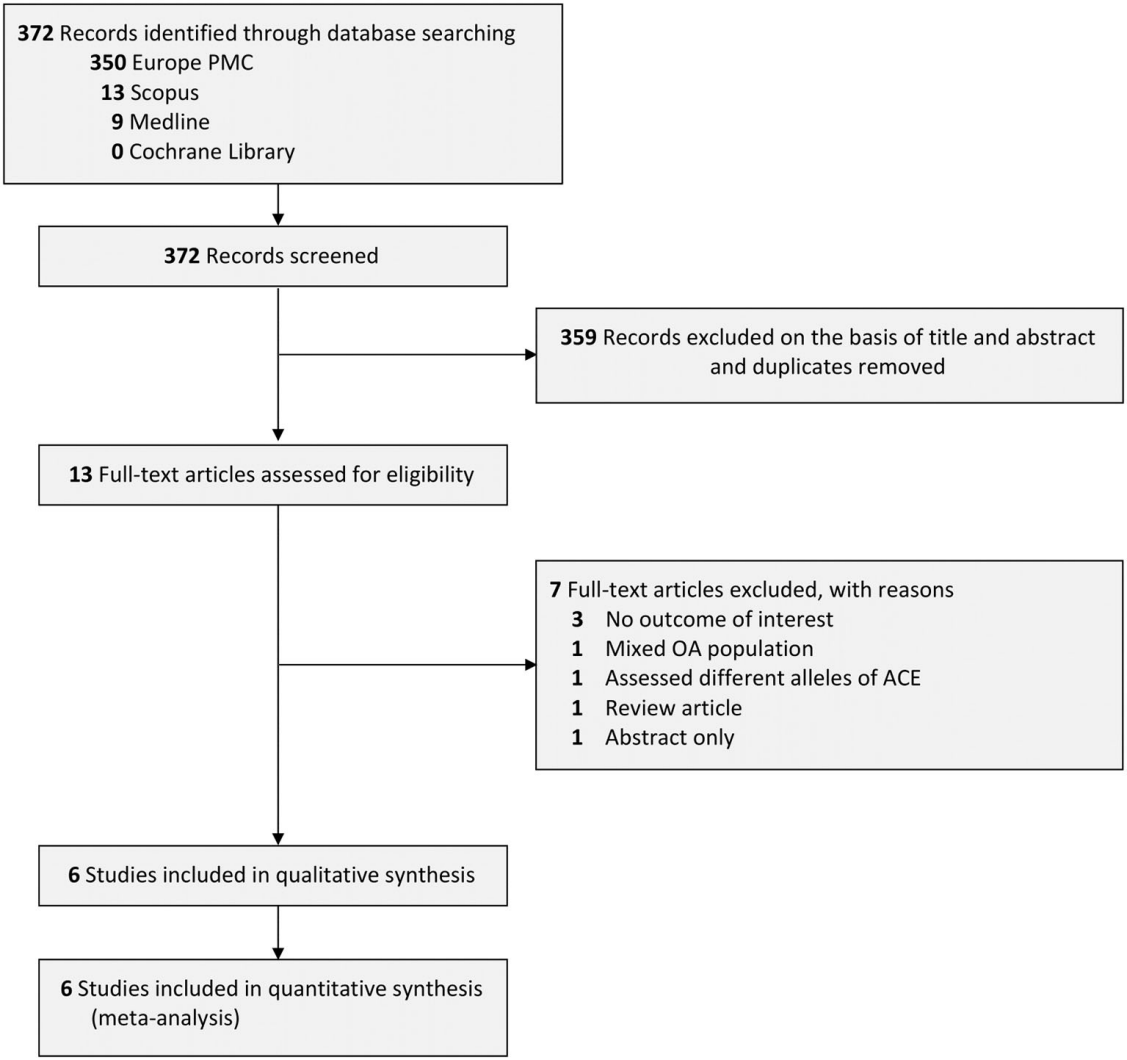
PRISMA diagram of the detailed process of selection of studies for inclusion in the systematic review and meta-analysis.

A literature search of the four databases yielded a total of 372 studies. After eliminating duplicates and screening articles based on their titles/abstracts, 359 studies were excluded and 13 were left for full-text assessment. Of these 13 studies, 7 studies were further excluded for the following reasons: 3 studies did not have the suitable outcome of interest data, 1 study involved a mixed OA population, 1 study conducted an assessment on different alleles of ACE, 1 study was a literature review, and 1 study was only available in abstract form. Ultimately, there were 6 studies ^11^^,^^12^^,^^16^^–^^19^ included in the final analysis with 1,226 patients with knee OA and 1,145 healthy subjects as controls ( Figure 1). All included studies had a case-control design. The countries of origin of each included study were China, Taiwan, South Korea, India, Turkey, and Kuwait. For analysis purposes, studies originating from China, Taiwan, South Korea, and India were grouped into the Asian ethnicity group while studies originating from Turkey and Kuwait were grouped into the Arab ethnicity group. Further details regarding the baseline characteristics of the included studies were summarized in Table 1.

**Table 1.** Characteristics of the included studies.

Study ID			Cases						Control			
Authors	Country	HWE test	Sample size	Age (mean ± SD)	Male (%)	BMI kg/m ^2^	Kellgren–Lawrence grade	Duration of OA	Sample size	Age (mean ± SD)	Male (%)	BMI kg/m ^2^
Bayram B et al. ^16^ 2011	Turkey	0.045	140	54.1 ± 1.2	37.2%	27.9 ± 0.2	I = 5.7% II = 12.9% III = 40% IV = 41.5%	N/A	60	44.6 ± 2	28.3%	25.2 ± 0.3
Chen G et al. ^17^ 2019	China	0.963	282	54.3 ± 4.5	26.6%	27.4 ± 3.3	I = 22% II = 50% III = 23% IV = 5%	7.5 ± 4.1	316	54.6 ± 5.4	24.3%	25 ± 3.4
Hong SJ et al. ^11^ 2003	South Korea	0.292	142	58.6 ± 9.4	33.8%	25.2 ± 3	I = 4.2% II = 55.6% III = 37.4% IV = 2.8%	6.6 ± 6.7	135	59.9 ± 8.5	32.6%	N/A
Lin C et al. ^18^ 2016	Taiwan	0.998	447	74.9 ± 7.1	43.4%	24.5 ± 3.3	II = 79.9% III = 19.4% IV = 0.7%	N/A	423	73.3 ± 6.6	48.7%	23.9 ± 3
Poornima S et al. ^19^ 2015	India	0.799	100	42.4 ± 8.1	32%	31.4 ± 3.4	N/A	2.9 ± 1.2	100	42.1 ± 7.9	31%	25.9 ± 2.6
Shehab DK et al. ^12^ 2008	Kuwait	<0.001	115	57.1 ± 9.1	11.3%	31.7 ± 6.4	I = 14.8% II = 38.3% III = 33% IV = 13.9%	5.9 ± 5	111	N/A	46.8%	N/A

BMI = body mass index, HWE = Hardy-Weinberg equilibrium, NA = not available, OA = osteoarthritis, SD = standard deviation.

### Quality of study assessment

Based on the results of the study quality assessment using the NOS tool, it was found that all included case-control studies had “good quality” so they were deemed worthy of being included in the meta-analysis. A summary of the assessment of study quality is presented in Table 2.

**Table 2.** Newcastle-Ottawa quality assessment of observational studies.

First author, year	Study design	Selection ^a^	Comparability ^b^	Outcome ^c^	Total score	Result
Bayram B et al. ^16^ 2011	Case-control	***	**	**	7	Good
Chen G et al. ^17^ 2019	Case-control	***	**	***	8	Good
Hong SJ et al. ^11^ 2003	Case-control	***	**	***	8	Good
Lin C et al. ^18^ 2016	Case-control	***	**	**	7	Good
Poornima S et al. ^19^ 2015	Case-control	***	**	**	7	Good
Shehab DK et al. ^12^ 2008	Case-control	***	**	**	7	Good

^a^ (1) representativeness of the exposed cohort; (2) selection of the non-exposed cohort; (3) ascertainment of exposure; (4) demonstration that outcome of interest was not present at start of study.

^b^ (1) comparability of cohorts on the basis of design or analysis, (maximum two stars).

^c^ (1) assessment of outcome; (2) was follow-up long enough for outcomes to occur; (3) adequacy of follow up of cohorts.

### Classical model

***Dominant (DD + ID vs. II)***

**Figure 2.**
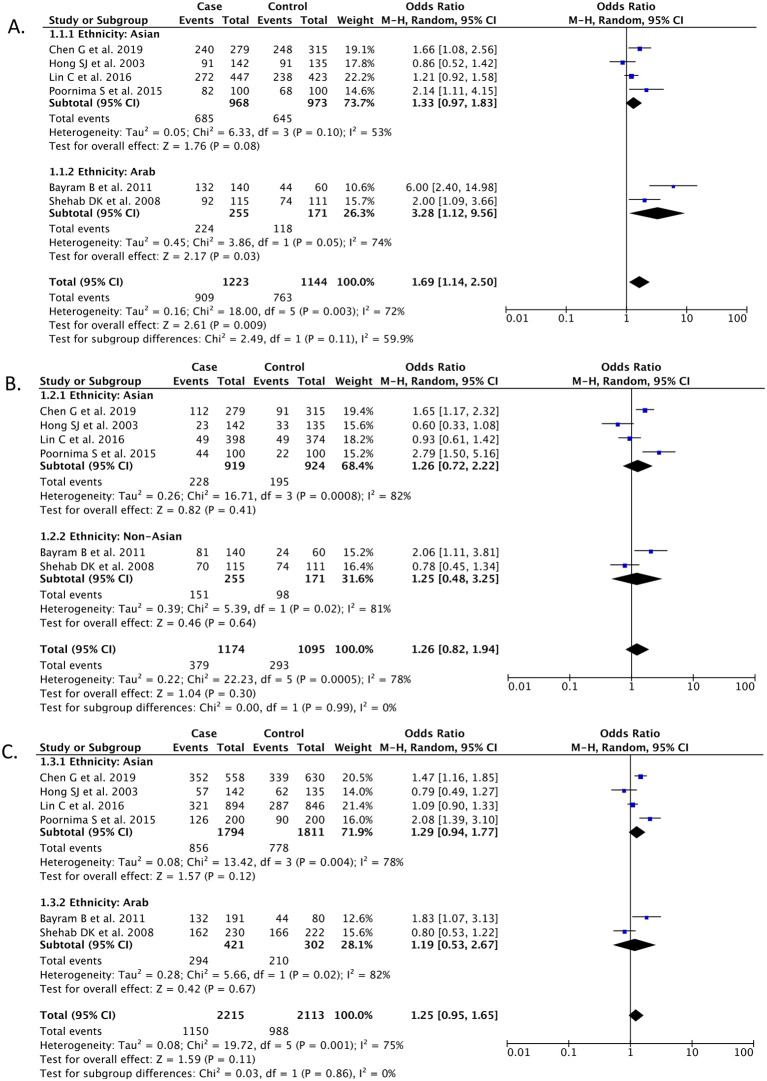
Forest plot that demonstrates the association between ACE gene I/D polymorphism and risk of knee OA in the dominant (DD + ID vs. II) (A), recessive (DD vs. ID + II) (B), and alleles (D vs. I) (C) models.

Based on our pooled analysis of 6 studies (n = 2,367), it has been shown that the dominant model of ACE gene I/D polymorphism (DD + ID vs. II) was associated with a higher risk of developing knee OA [OR 1.69 (95% CI 1.14 – 2.50), *p* = 0.009, *I* ^2^ = 72%, random-effect models] ( Figure 2A). However, subgroup analysis based on ethnicity revealed that a statistically significant association was only observed in the Arab ethnicity subgroup ( *p* = 0.03), but not in the Asian ethnicity subgroup ( *p* = 0.08) ( Figure 2A).

***Recessive (DD vs. ID + II)***

Based on our pooled analysis of 6 studies (n = 2,269), it has been shown that the recessive model of ACE gene I/D polymorphism (DD vs. ID + II) was not associated with knee OA risk [OR 1.26 (95% CI 0.82 – 1.94), *p* = 0.30, *I* ^2^ = 78%, random-effect models] ( Figure 2B). A non-significant association was also observed both in the Asian ethnicity ( *p* = 0.41) and Arab ethnicity subgroups ( *p =* 0.64) ( Figure 2B).

***Alleles (D vs. I)***

Our meta-analysis from a total of 6 studies (n = 4,328) showed that neither alleles of ACE gene I/D polymorphism (D vs. I) was associated with the risk of knee OA [OR 1.25 (95% CI 0.95 – 1.65), *p* = 0.11, *I* ^2^ = 75%, random-effect models] ( Figure 2C). A non-significant association was also observed both in the Asian ethnicity ( *p* = 0.12) and Arab ethnicity subgroups ( *p =* 0.67) ( Figure 2C).

### Additional model

***DD vs. ID***

**Figure 3.**
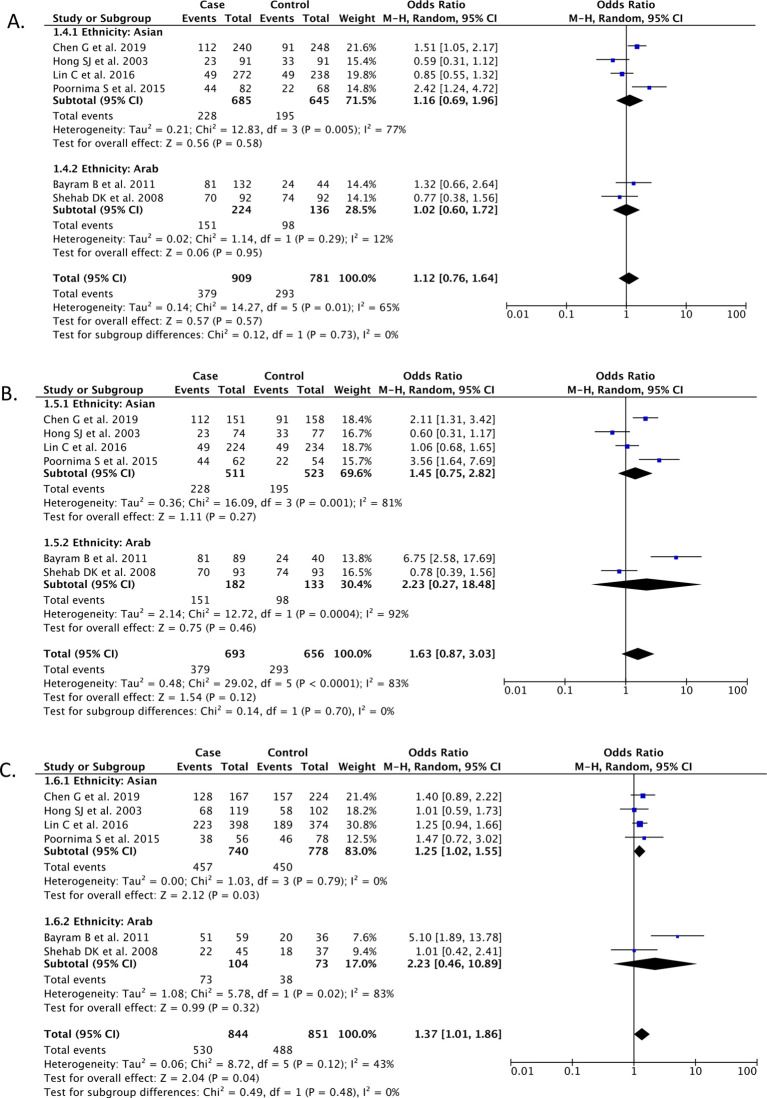
Forest plot that demonstrates the association between ACE gene I/D polymorphism and risk of knee OA in the DD vs. ID (A), DD vs. II (B), and ID vs. II models.

Our meta-analysis from a total of 6 studies (n = 1,690) showed that the genetic model comparing DD with ID genotypes of ACE gene I/D polymorphism was not associated with the risk of knee OA [OR 1.12 (95% CI 0.76 – 1.64), *p* = 0.57, *I* ^2^ = 65%, random-effect models], and the results remained consistent for both subgroups of ethnicity ( *p* for Asian = 0.58; *p* for Arab = 0.95) ( Figure 3A).

***DD vs. II***

Our meta-analysis from a total of 6 studies (n = 1,349) showed that the genetic model comparing DD with II genotypes of ACE gene I/D polymorphism was not associated with the risk of knee OA [OR 1.63 (95% CI 0.87 – 3.03), *p* = 0.12, *I* ^2^ = 83%, random-effect models], and the results remained consistent for both subgroups of ethnicity ( *p* for Asian = 0.27; *p* for Arab = 0.46) ( Figure 3B).

***ID vs. II***

Our meta-analysis from a total of 6 studies (n = 1,349) showed that the genetic model involving ID and II genotypes of ACE gene I/D polymorphism was associated with a higher risk of knee OA [OR 1.37 (95% CI 1.01 – 1.86), *p* = 0.04, *I* ^2^ = 43%, random-effect models], but the results remained significant only for Asian ethnicity subgroup ( *p* = 0.03), and not for Arab ethnicity subgroups ( *p* = 0.32) ( Figure 3C).

### Meta-regression

The summary of the results from the meta-regression analysis of several factors that may influence the relationship between ACE gene I/D polymorphism and knee OA is summarized in Table 3. From the results of the meta-regression analysis, it was found that ethnicity (Asian as reference) ( *p* = 0.0461) has a significant effect on the relationship between ACE gene I/D polymorphism and knee OA risk in the dominant model (DD + ID vs. II). In addition, our regression analysis also showed that sex is an important variable that significantly influences the risk of knee OA in 3 genetic models of ACE gene I/D polymorphism, namely recessive (DD vs. ID + II) ( *p* = 0.0378), alleles (D vs. I) ( *p* = 0.0060), and DD vs. DI models ( *p* = 0.0332). Other factors, such as sample size from included studies, age, and BMI did not significantly influence the relationship between ACE gene I/D polymorphism and the risk of knee OA.

**Table 3.** Results for the meta-regression for each outcome of interest.

Dominant (DD + ID vs. II)					
Covariate	Coefficient	95% CI (min)	95% CI (max)	S.E.	*p-*value
**Ethnicity (Asian as reference)**	**0.8181**	**0.0140**	**1.6221**	**0.4102**	**0.0461**
Sample size (number)	-0.0005	-0.0026	0.0015	0.0010	0.6007
Age (years)	-0.0179	-0.0739	0.0380	0.0286	0.5304
Sex (Male prevalence)	0.0041	-0.0479	0.0560	0.0265	0.8782
BMI (kg/m ^2^)	0.1055	-0.0571	0.2682	0.0830	0.2035
**Recessive (DD vs. ID + II)**					
Ethnicity (Asian as reference)	0.0104	-1.1046	1.1255	0.5689	0.9854
Sample size (number)	0.0005	-0.0016	0.0025	0.0010	0.6433
Age (years)	0.0136	-0.0406	0.0678	0.0276	0.6225
**Sex (Male prevalence)**	**0.0381**	**0.0021**	**0.0741**	**0.0184**	**0.0378**
BMI (kg/m ^2^)	-0.0337	-0.2220	0.1545	0.0960	0.7256
**Allele (D vs. I)**					
Ethnicity (Asian as reference)	-0.0867	-0.8376	0.6641	0.3831	0.8209
Sample size (number)	0.0004	-0.0009	0.0017	0.0007	0.5625
Age (years)	0.0091	-0.0272	0.0453	0.0185	0.6236
**Sex (Male prevalence)**	**0.0284**	**0.0081**	**0.0487**	**0.0103**	**0.0060**
BMI (kg/m ^2^)	-0.0355	-0.1584	0.0874	0.0627	0.5709
**DD vs. ID**					
Ethnicity (Asian as reference)	-0.1352	-1.0896	0.8192	0.4869	0.7813
Sample size (number)	0.0006	-0.0012	0.0023	0.0009	0.5264
Age (years)	0.0206	-0.0237	0.0649	0.0226	0.3615
**Sex (Male prevalence)**	**0.0352**	**0.0028**	**0.0676**	**0.0165**	**0.0332**
BMI (kg/m ^2^)	-0.0453	-0.2060	0.1154	0.0820	0.5806
**DD vs. II**					
Ethnicity (Asian as reference)	0.3872	-1.3016	2.0761	0.8617	0.6531
Sample size (number)	0.0001	-0.0031	0.0033	0.0016	0.9577
Age (years)	-0.0075	-0.0930	0.0780	0.0436	0.8627
Sex (Male prevalence)	0.0491	-0.0130	0.1111	0.0317	0.1214
BMI (kg/m ^2^)	0.0189	-0.2744	0.3121	0.1496	0.8997
**ID vs. II**					
Ethnicity (Asian as reference)	0.4857	-0.2019	1.1732	0.3508	0.1662
Sample size (number)	-0.0003	-0.0013	0.0007	0.0005	0.5786
Age (years)	-0.0170	-0.0500	0.0159	0.0168	0.3116
Sex (Male prevalence)	0.0141	-0.0165	0.0447	0.0156	0.3670
BMI (kg/m ^2^)	0.0489	-0.0507	0.1485	0.0508	0.3356

### Publication bias

The number of studies for each outcome of interest in this review is less than 10 studies where funnel plots and statistical tests to detect publication bias are less reliable, ^20^^,^^21^ so publication bias analysis was not performed in this study.

## Discussion

Based on the results of our meta-analysis, it has been shown that the genetic models of ACE gene I/D polymorphism that play a significant role in the risk of knee OA were only seen in the dominant (DD + ID vs. II) model for Arab ethnicity population and ID vs. II models for Asian ethnicity population. Other genetic models of ACE gene I/D polymorphism, such as recessive (DD vs. ID + II), alleles (D vs. I), DD vs. ID, and DD vs. II models did not show a significant relationship with the risk of knee OA, both in Asian ethnicity and Arab ethnicity subgroups. Further regression analysis also showed that ethnicity and sex were variables that significantly influenced the risk of knee OA in several genetic models.

The results of our meta-analysis are similar to the previous meta-analysis written by Lin C et al. ^18^ In their meta-analysis of 6 studies, Lin C et al. ^18^ showed that the relationship between ACE gene I/D polymorphism and knee OA were only seen in the dominant (DD + ID vs. II) and ID vs. II genetic models. Other genetic models did not show a significant relationship with knee OA risk. Nonetheless, there are some differences between the meta-analyses by Lin C et al. ^18^ with our current meta-analysis.

First, although the total number of included studies is the same (6 studies), Lin C et al. ^18^ have mistakenly included the study by Inanir A et al. ^22^ which includes not only the population of patients with knee OA but also patients with hip and hand OA. This of course contradicts the title and eligibility criteria of the study by Lin C et al. ^18^ which stated that only knee OA studies would be included in the analysis. In addition, including studies with different populations of patients (patients with osteoarthritis other than knee) is inappropriate according to the Cochrane Handbook for Systematic Review because it will also have an impact on the increased heterogeneity between studies. ^23^ On the other hand, we fully adhere to the Cochrane guidelines where only studies with knee OA population were included in the analysis and discarded the study by Inanir A et al. ^22^ Instead, we included 1 additional study by Chen G et al. ^17^ published in 2019 which was not included in the previous meta-analysis by Lin C et al. ^18^

Second, the previous meta-analysis by Lin C et al. ^18^ involved 4 variables namely ethnicity, sex, age, and BMI in the meta-regression analysis. However, the sex variable was only analyzed as a categorical moderator (female or male) and the age and BMI variables were only calculated based on a certain number of multiplications (every 10 years for age and every 5 kg/m ^2^ for BMI). ^18^ This certainly can have an impact on the lack of accuracy within the results from the meta-regression analysis of this variable. In contrast, in our meta-regression analysis, the variable sex was calculated as male sex prevalence and the variable age and BMI were not calculated based on multiples of certain numbers but based on their true values so that any addition or reduction in the value of the variable, even it is small, is still meaningful. By doing this, we have found that sex influenced the relationship between ACE gene I/D polymorphism and knee OA risk in recessive (DD vs. ID + II), alleles (D vs. I), and DD vs. ID genetic models, a result not found in the previous meta-analysis. Our study also added the sample size variable to the regression analysis so that a total of 5 variables were analyzed further.

Our study is certainly not without some limitations. Notable heterogeneities were identified in some of the genetic models in our study, even after subgroup analysis based on ethnicity, which is difficult to explain. The total number of studies included in our analysis is also relatively small due to limited evidence regarding ACE gene I/D polymorphism on knee OA risk. The majority of the included studies also come from Asian countries with populations of Asian ethnicity and only a portion of the studies involve populations of Arab ethnicity. Data regarding other ethnicities, such as Caucasian, Hispanic, and Black so far cannot be found so they cannot be analyzed further, and thus, the results from this study are also not necessarily applicable to those ethnicities.

## Conclusion

Our systematic review and meta-analysis suggest that only the dominant (DD + ID vs. II) model of ACE gene I/D polymorphism in Arab populations and the ID vs. II models of ACE gene I/D polymorphism in Asian populations have a significant association with a higher risk of knee OA. Other genetic models of ACE gene I/D polymorphism, including recessive (DD vs. ID + II), alleles (D vs. I), DD vs. ID, and DD vs. II were not associated with the risk of knee OA. Further studies with larger sample sizes and involving different ethnic groups are still needed to confirm the results of our study.

### Registration and protocol

This review was registered on PROSPERO ID CRD42023398472 (Association between Angiotensin-Converting Enzyme (ACE) gene I/D polymorphism with the risk of knee OA).

## Data Availability

All data underlying the results are available as part of the article and no additional source data are required. Data analyzed in this study were a re-analysis of existing data, which are openly available at locations cited in the reference section. Figshare: PRISMA_2020_abstract_checklist.pdf, https://doi.org/10.6084/m9.figshare.25019756.v2. ^24^ This project contains the following extended data:
• repository.xlsx (characteristics of all articles included in the research; forest plot data for Figures 2 and 3). Figshare: PRISMA checklist for ‘Association between angiotensin-converting enzyme (ACE) gene I/D polymorphism with the risk of knee OA: A systematic review, meta-analysis, and meta-regression’, https://doi.org/10.6084/m9.figshare.25019756.v2. ^24^

## References

[1] Sharma L : Osteoarthritis of the Knee. *N. Engl. J. Med.* 2021 Jan 7;384(1):51–59. 10.1056/NEJMcp1903768 33406330

[2] Cui A Li H Wang D : Global, regional prevalence, incidence and risk factors of knee osteoarthritis in population-based studies. *EClinicalMedicine.* 2020 Nov 26;29-30:100587. 10.1016/j.eclinm.2020.100587 34505846 PMC7704420

[3] Silverwood V Blagojevic-Bucknall M Jinks C : Current evidence on risk factors for knee osteoarthritis in older adults: a systematic review and meta-analysis. *Osteoarthr. Cartil.* 2015 Apr;23(4):507–515. 10.1016/j.joca.2014.11.019 25447976

[4] Fernández-Moreno M Rego I Carreira-Garcia V : Genetics in osteoarthritis. *Curr. Genomics.* 2008 Dec;9(8):542–547. 10.2174/138920208786847953 19516961 PMC2694558

[5] Chang Y Wei W : Angiotensin II in inflammation, immunity and rheumatoid arthritis. *Clin. Exp. Immunol.* 2015 Feb;179(2):137–145. 10.1111/cei.12467 25302847 PMC4298392

[6] De Falco L Fioravanti A Galeazzi M : Bradykinin and its role in osteoarthritis. *Reumatismo.* 2013 Jul 23;65(3):97–104. 10.4081/reumatismo.2013.97 23884024

[7] Meini S Maggi CA : Knee osteoarthritis: a role for bradykinin? *Inflamm. Res.* 2008 Aug;57(8):351–361. 10.1007/s00011-007-7204-1 18787773

[8] Wu Y Lu X Li M : Renin-angiotensin system in osteoarthritis: A new potential therapy. *Int. Immunopharmacol.* 2019 Oct;75:105796. 10.1016/j.intimp.2019.105796 31408841

[9] Yigit S Inanir A Tural S : Association of angiotensin converting enzyme (ACE) gene I/D polymorphism and rheumatoid arthritis. *Gene.* 2012 Dec 10;511(1):106–108. 10.1016/j.gene.2012.09.036 23000565

[10] Uppal SS Haider MZ Hayat SJ : Significant association of insertion/deletion polymorphism of the angiotensin-converting enzyme gene with rheumatoid arthritis. *J. Rheumatol.* 2007 Dec;34(12):2395–2399. 17985406

[11] Hong SJ Yang HI Yoo MC : Angiotensin converting enzyme gene polymorphism in Korean patients with primary knee osteoarthritis. *Exp. Mol. Med.* 2003 Jun 30;35(3):189–195. 10.1038/emm.2003.26 12858018

[12] Shehab DK Al-Jarallah KF Alawadhi AM : Prevalence of angiotensin-converting enzyme gene insertion-deletion polymorphism in patients with primary knee osteoarthritis. *Clin. Exp. Rheumatol.* 2008 Mar-Apr;26(2):305–310. 18565253

[13] Page MJ McKenzie JE Bossuyt PM : The PRISMA 2020 statement: an updated guideline for reporting systematic reviews. *BMJ.* 2021 Mar 29;372:n71. 10.1136/bmj.n71 33782057 PMC8005924

[14] Stang A : Critical evaluation of the Newcastle-Ottawa scale for the assessment of the quality of nonrandomized studies in meta-analyses. *Eur. J. Epidemiol.* 2010 Sep;25(9):603–605. 10.1007/s10654-010-9491-z 20652370

[15] Higgins JP Thompson SG Deeks JJ : Measuring inconsistency in meta-analyses. *BMJ.* 2003 Sep 6;327(7414):557–560. 10.1136/bmj.327.7414.557 12958120 PMC192859

[16] Bayram B Sayın E Güneş HV : DD genotype of ace gene I/D polymorphism is associated in a Turkish study population with osteoarthritis. *Mol. Biol. Rep.* 2011 Mar;38(3):1713–1716. 10.1007/s11033-010-0284-y 20845076

[17] Chen G Hu S Lai Z : Association between ACE gene I/D polymorphism and knee osteoarthritis in a Chinese population. *Biosci. Rep.* 2019 Feb 26;39(2):BSR20181713. 10.1042/BSR20181713 30765613 PMC6390131

[18] Lin C Chen HC Fang WH : Angiotensin-Converting Enzyme Insertion/Deletion Polymorphism and Susceptibility to Osteoarthritis of the Knee: A Case-Control Study and Meta-Analysis. *PLoS One.* 2016 Sep 22;11(9):e0161754. 10.1371/journal.pone.0161754 27657933 PMC5033346

[19] Poornima S Subramanyam K Khan IA : The insertion and deletion (I28005D) polymorphism of the angiotensin I converting enzyme gene is a risk factor for osteoarthritis in an Asian Indian population. *J. Renin-Angiotensin-Aldosterone Syst.* 2015 Dec;16(4):1281–1287. 10.1177/1470320314547403 25178458

[20] Thornton A Lee P : Publication bias in meta-analysis: its causes and consequences. *J. Clin. Epidemiol.* 2000 Feb;53(2):207–216. 10.1016/s0895-4356(99)00161-4 10729693

[21] Terrin N Schmid CH Lau J : Adjusting for publication bias in the presence of heterogeneity. *Stat. Med.* 2003 Jul 15;22(13):2113–2126. 10.1002/sim.1461 12820277

[22] Inanir A Yigit S Tural S : MTHFR gene C677T mutation and ACE gene I/D polymorphism in Turkish patients with osteoarthritis. *Dis. Markers.* 2013;34(1):17–22. 10.3233/DMA-2012-00939 23089924 PMC3809745

[23] Higgins JPT Thomas J Chandler J : *Cochrane Handbook for Systematic Reviews of Interventions version 6.3 (updated February 2022).* Cochrane;2022. Reference Source

[24] Rasyid H : PRISMA_2020_abstract_checklist.pdf.[Dataset]. *figshare.* 2024. 10.6084/m9.figshare.25019756.v2

