# Supplementary material for: Association between angiotensin-converting enzyme (ACE) gene I/D polymorphism with the risk of knee OA: A systematic review, meta-analysis, and meta-regression
Source: F1000Res. 2024 Feb 26;13:146. [Version 1] doi: 10.12688/f1000research.140233.1 (PMC11109534; doi:10.12688/f1000research.140233.1)
Supplement: Peer review history

# Reviewer report: Reviewer response for version 1

**DOI:** 10.5256/f1000research.153567.r252579

**Published:** 2024-05-10

**Authors:** Yoyos Dias Ismiarto

**Affiliations:** 1 Padjadjaran University, Bandung, Indonesia

Major comment:

 The study's results are significant in that they contribute to the understanding of the genetic factors involved in the development of knee OA and may have implications for the prevention and treatment of the disease. However, there are some data that need clarification: 

 1. **Sample Size Discrepancy**: The total number of samples is reported as 2,371 in the study selection and characteristics subsection, but as 2,367 in the classical model subsection. 

  2. **Focus on Results and Implications**: The authors should also explore how factors interact with the ACE gene I/D polymorphism to influence the outcomes of knee OA. This would provide a more comprehensive understanding of the genetic and environmental factors involved in the disease. 

 Minor comment:

 This manuscript presents a comprehensive analysis of the relationship between the angiotensin-converting enzyme (ACE) gene I/D polymorphism and the risk of knee osteoarthritis (OA). The meta-analysis was well written according to the PRISMA guidelines. The authors also provide a comprehensive summary of the existing literature on the topic, highlighting the strengths and limitations of previous studies

Are the rationale for, and objectives of, the Systematic Review clearly stated?

Yes

Is the statistical analysis and its interpretation appropriate?

Yes

Are sufficient details of the methods and analysis provided to allow replication by others?

Yes

Are the conclusions drawn adequately supported by the results presented in the review?

Yes

Reviewer Expertise:

Orthopedics; Arthritis; Degenerative Disease; Osteoarthritis; Traumatology

I confirm that I have read this submission and believe that I have an appropriate level of expertise to confirm that it is of an acceptable scientific standard.

# Reviewer report: Reviewer response for version 1

**DOI:** 10.5256/f1000research.153567.r252578

**Published:** 2024-04-29

**Authors:** Nata Pratama Hardjo Lugito

**Affiliations:** 1 Pelita Harapan University, Tangerang, Indonesia

The systematic review, meta-analysis, and meta-regression manuscript was well written according to the PRISMA guidelines.

 There are some data that need clarification.

1. The total number of sample. In the result section, study selection and characteristics subsection it was written that the sample was 1,226 patients with knee OA and 1,145 healthy subjects as controls, making a total of 2,371. In the result section, classical model subsection, Dominant (DD + ID vs. II) it was written that the total number of sample was 2,367. Which one is the correct number?
2. In the figure 2B, the Arab ethnicity was written as Non-Asian ethnicity. Please correct

In the discussion, the authors mentioned that their study’s results was similar to the previous meta-analysis written by Lin C et al. Yet the authors, explained the difference between their study compared to Lin’s study. The authors should explain how the differences could still resulted in similar results.

 The authors should put more emphasise on their results and discuss the impact of ACE gene I/D polymorphism on the pathogenesis, therapy, and prognosis of knee OA, rather than making critical analysis on Lin’s study.

 The authors should also try to explain how ethnicity, age, sex, BMI, Kellgren-Lawrence grade, and duration of OA interact with the ACE gene I/D polymorphism.

Are the rationale for, and objectives of, the Systematic Review clearly stated?

Yes

Is the statistical analysis and its interpretation appropriate?

Yes

Are sufficient details of the methods and analysis provided to allow replication by others?

Yes

Are the conclusions drawn adequately supported by the results presented in the review?

Partly

Reviewer Expertise:

Internal medicine, clinical epidemiology

I confirm that I have read this submission and believe that I have an appropriate level of expertise to confirm that it is of an acceptable scientific standard.
